# Meta-synthesis of qualitative studies to explore fathers’ perspectives of their influence on children’s obesity-related health behaviors

**DOI:** 10.1186/s12912-024-01728-z

**Published:** 2024-01-30

**Authors:** Eunyoung Park, Myoungock Jang, Mi Sook Jung, Nondumiso Satiso Dlamini

**Affiliations:** College of Nursing, Chungnam National University, Munhwa-ro 266, Jung-gu, Daejeon, 35015 South Korea

**Keywords:** Fathers, Role model, Children’s health behaviors, Parenting practice, Family relationship, Meta-synthesis

## Abstract

**Background:**

In nursing research and practice, there is a paucity of information about how fathers perceive their role in shaping their children’s health behaviors. Most studies on the parental factors affecting children’s health behaviors have focused on the role of mothers. However, recent studies showed that fathers’ health behaviors can influence those of their children. Therefore, the aim of this study was to synthesize existing qualitative studies to explore fathers’ perspectives regarding how they influence children’s obesity-related health behaviors.

**Methods:**

We conducted a descriptive meta-synthesis. To retrieve relevant articles, we used databases including PubMed, CINAHL, and Web of Science. Only qualitative studies published in English-language peer-reviewed journals, targeting fathers of children aged 2–18 years, and focusing on fathers’ perspectives were included. All the quotes collected from the studies were reviewed and coded, and thematic analysis was used to derive themes.

**Results:**

Article screening and review yielded a total of 13 qualitative studies, from which the following themes emerged: (1) fathers’ parenting practices and role-modeling behaviors, (2) fathers’ roles in their relationships with their family members, and (3) fathers’ resource-seeking behaviors and contributions to their home food environment. Fathers were aware that their parenting practices and role-modeling behaviors could influence their children’s health behaviors. Furthermore, fathers recognized the importance of their relationships with family members, which was reflected in their family roles; that is, whether they took responsibility for childcare and household work, whether their parenting practices were similar to those of their spouses, and whether they involved their children in their activities. Fathers also reported their resource-seeking behaviors as well as their contribution to the home food environment, which affected their children’s health behaviors.

**Conclusion:**

Fathers’ perspectives on their influence on children’s health behaviors reveal their unique paternal role in influencing children’s health behaviors. Fathers’ perspectives could be incorporated into future nursing research to examine the relationship between fathers’ roles and children’s health behaviors to develop better health intervention programs.

**Supplementary Information:**

The online version contains supplementary material available at 10.1186/s12912-024-01728-z.

## Introduction

Childhood obesity poses a threat to global public health. Numerous interventions have been developed and implemented to combat childhood obesity with mixed short-term effects [1, 2]. Multiple factors influence children’s health behaviors, including diet, physical activity, and sedentary behavior; among these, parents play a significant role in shaping their children’s health behaviors. Investigations of parental influence on children’s health behaviors have revealed multiple factors and their relationships with children’s health behaviors [3, 4].

Parental influence on children’s health behaviors is often assessed in terms of parents’ parenting practices, which comprise their behavioral strategies for interacting with their children [5]. Parenting practices, including applying specific rules to reach a compromise with their children, such as monitoring and setting limits on specific behaviors, can promote healthy behaviors in children [6, 7]. Conversely, parenting practices involving stringent rules to control children’s behaviors without a prior discussion or compromise [8] or those involving excessive permissiveness or minimal rules may promote unhealthy behaviors among children [9].

Despite the scholarly consensus on parental influence on children’s health behaviors, a notable issue with such investigations is that they have mainly focused on the role of mothers or female guardians [10]. A systematic review of observational studies found that fathers were less likely to participate in research on parenting practices related to childhood obesity [11]. Furthermore, fathers were involved in a limited number of intervention programs designed to improve their children’s health behaviors and address childhood obesity [12].

There may be some reasons for the underrepresentation of fathers in such studies. Traditionally, parents’ roles were more clearly divided, as the mother took primary responsibility for childcare, whereas the father took the role of the breadwinner [12]. The differences in the socially constructed roles of fathers and mothers may have influenced fathers’ research participation. Mothers commonly consider themselves the main caregivers of their children; thus, they may tend to participate in such research more actively. However, while this rationale may apply to traditional families, family dynamics have changed over time, and family roles have become more complicated [13]. For instance, both parents often work outside the home and share childcare responsibilities; thus, the mother is no longer the only main caregiver [14]. Consequently, an approach that mainly targets mothers may be limited in capturing the dynamics of parental influence on children’s health behaviors. Consistent with this assumption, it was found that the parent’s sex is a possible moderator for understanding parental influence on children’s health behaviors and obesity in a systematic review of observational studies [15].

Emerging evidence suggests that fathers’ unhealthy feeding practices predict the increase in children’s body weight and that fathers’ diet quality is positively associated with the overall quality of children’s food consumption [16]. Notably, one study reported that the father’s weight had a greater impact on the likelihood of children being obese than the mother’s weight [17]. Despite the potential influence of fathers on their children’s health behaviors and weight, the literature on nursing research and practice lacks relevant information on ways to mitigate fathers’ potential negative impact on children’s health behaviors. Nevertheless, several qualitative studies have explored fathers’ parenting, attitudes, perceptions, and roles in children’s health behaviors [18–20]. Synthesizing previous qualitative studies would provide a more in-depth understanding of how fathers perceive their influence on their children’s health behaviors. A qualitative meta-synthesis is the systematic integration of results from qualitative studies to provide a more comprehensive exploration of the complex phenomenon [21]. Therefore, this study aimed to synthesize qualitative results to explore fathers’ perspectives regarding how they influence children’s obesity-related health behaviors. The research question was: What are fathers’ perspectives about their influence on children’s health behaviors related to childhood obesity?

## Methods

We utilized the qualitative meta-synthesis methodology to synthesize the outcomes of qualitative studies that explored fathers’ perspectives on their influence on their children’s health behaviors. This research was performed in accordance with the Enhancing transparency in reporting the synthesis of qualitative research (ENTREQ) guideline [22] and the Preferred Reporting Items for Systematic Reviews and Meta-Analyses (PRIMSA) guideline [23].

### Identification and selection of relevant articles

The relevant articles were identified by retrieving articles from database searches and manual searching for other sources, including review articles identified through the initial search. We searched the PubMed, Cumulative Index to Nursing and Allied Health Literature, and Web of Science databases using combinations of the following search terms: (*father* OR *paternal*) AND (*role* OR *perspective*) AND (*children* OR *child*) AND (*health behavior* OR *eating* OR *physical activity* OR *sedentary behavior*). We did not use search terms pertaining to the study design because they may limit the relevant literature search.

We included qualitative research articles that met the following criteria: 1) the study focused on fathers’ perspectives on their influence on children’s health behaviors; 2) the study targeted fathers of children aged 2–18 years; and 3) the article was written in English and published in a peer-reviewed journal published by July 2023. We excluded articles based on the following criteria: 1) the study used a study design other than a qualitative study design; 2) the study included both parents and did not differentiate between the outcomes derived from each parent’s statements; and 3) the study targeted the families of children with health conditions and their influence on these children’s eating behaviors or physical activities. We also excluded articles on other people’s (e.g., the mother or healthcare providers) reports on fathers’ influence on children’s health behaviors because we solely focused on fathers’ perspectives. Finally, we did not include studies focusing on prevention or management of childhood obesity because we aimed to explore fathers’ perspectives on their influence on children’s health behaviors rather than their efforts to improve their children’s behaviors.

After importing all the articles into EndNote, author M.J., who has experience with systematic review studies, performed the initial screening by deleting duplicate articles and excluding non-qualitative studies and non-English articles. After the initial screening, author M.J. and co-author N.D. (a trained graduate student), independently reviewed the abstracts and full texts, if necessary, for further screening. Subsequently, the two co-authors compared their screening results and reached a consensus through discussion of any discrepancies.

### Quality assessment

We performed a quality assessment using the Checklist for Qualitative Research, which is a part of the Joanna Briggs Institute’s Critical Appraisal tools [24]. This checklist helps assess the methodological quality of a qualitative study and determine the extent to which the study has addressed the possibility of bias in its design, conduct, and analysis. This assessment tool comprises 10 questions regarding the quality of any type of qualitative research, with responses such as “yes,” “no,” “unclear,” and “not applicable.” To perform the quality appraisal assessment, the two co-authors (M.J. and N.D.) independently assessed the quality of each study based on each question, compared their results, and discussed any incongruence to reach a consensus. This assessment tool does not have specific guidelines on grading or cut-off points for inclusion. Thus, we calculated the score by assigning one point to each question, resulting in a total score of 10.

### Data extraction and synthesis

We utilized the data extraction and synthesis methods described by Sandelowski and Barroso [25]. According to these scholars, the goal of this meta-synthesis is to summarize and re-analyze the findings from each study to derive the main themes. Our goal in synthesizing the included qualitative studies was to find fathers’ perspectives that were the most indicative of their influence on children’s health behaviors.

In this process, we created a data matrix to record the extracted articles. The matrix included aspects such as the study design, geographic area, sample characteristics (sample size, interviewees’ age range, children’s age range, race, and ethnicity), data collection methods, and data analysis methods. The co-authors (E.P. and N.D.) extracted all the quotes and themes from each study. Of the studies that included data on both parents, only quotes made by the fathers were retained [26, 27].

All the extracted quotes were inputted onto an Excel spreadsheet and re-coded. Although the responses could differ depending on the data collection approach used (i.e., interviews or focus groups), we depended solely on the data presented in primary studies. All the quotes—and themes supported by the quotes—were derived from the primary studies included in the meta-synthesis. The data analysis was conducted using a thematic approach. Two co-authors (E.P. and M.J.) with experience and expertise in qualitative research independently coded the quotes and reached an agreement through thorough comparisons of the codes and frequent discussions. If the coders noticed any discrepancy in specific codes, they checked how specific quotes were used to support the themes in the primary studies and then reached consensus. After all the data were coded, they were iteratively reviewed and collapsed into categories with similar meanings. Furthermore, themes and overarching themes were derived.

## Results

### Study selection

**Fig. 1:**
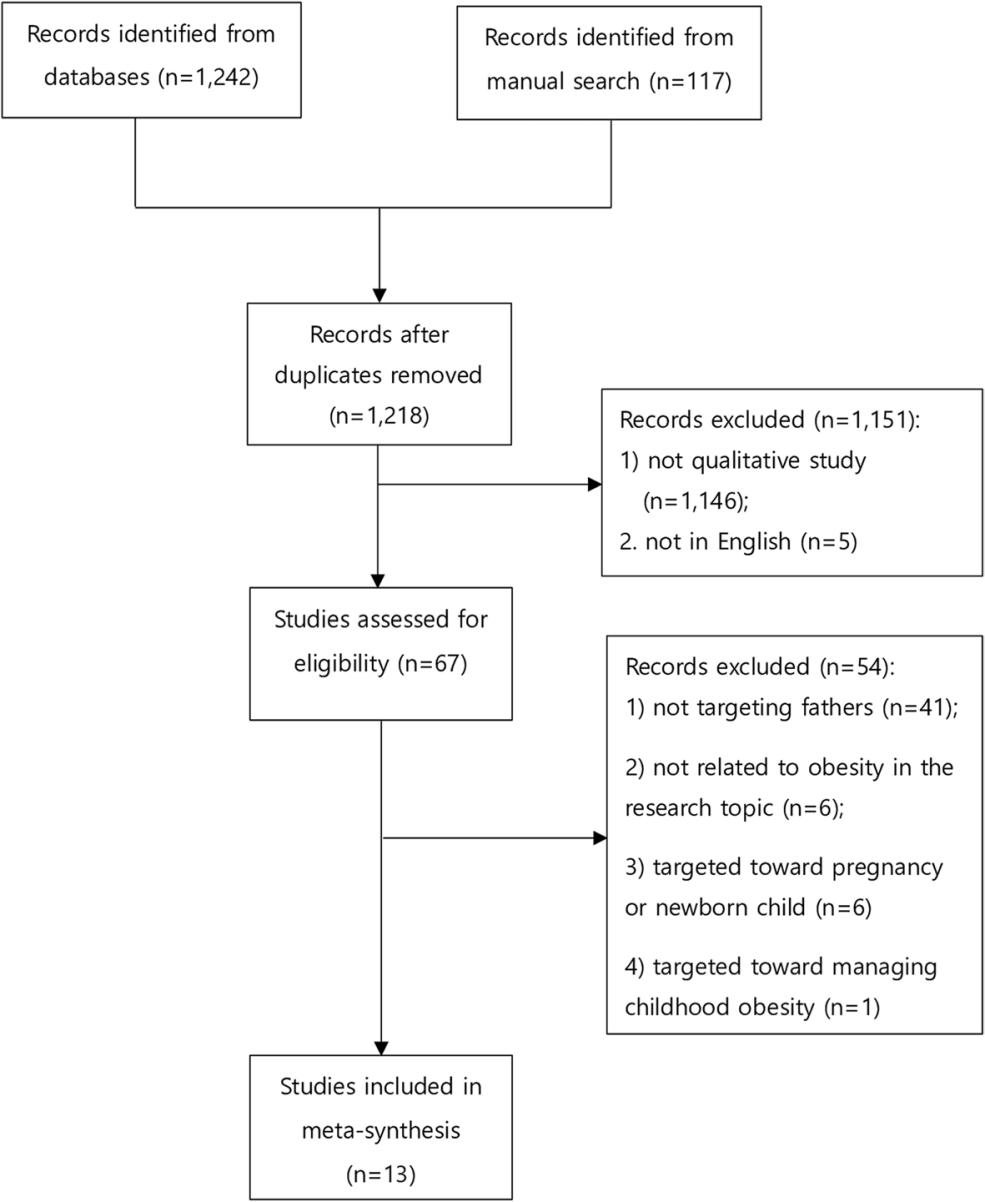
Flow of the literature searching and screening

Database and manual searches yielded 1,242 and 117 studies, respectively. After the initial screening, 1,151 studies were excluded. After a full-text screening of 67 studies, we further excluded 54 studies for the following reasons: they did not target fathers (*n* = 41), did not focus on children’s health behaviors related to obesity (*n* = 6), did not target children aged 2–18 years (*n* = 6), or targeted fathers’ experience of weight management of children (*n* = 1). Finally, 13 studies were included in this qualitative meta-analysis. The article selection process is illustrated in Fig. 1.

### Study description

Among the 13 qualitative studies, the sample sizes ranged from 8 to 117, and the data were collected through individual interviews, focus groups, or surveys with open-ended questions. Participants were recruited using either convenience sampling or purposive sampling methods. In one study, fathers were recruited from the worksite [28], and in two, fathers were recruited from specific ethnic communities [27, 29]. In two studies, fathers and mothers participated in focus group interviews [26, 27], whereas in the remaining studies, only fathers participated in individual interviews. The target age of the children varied across the studies but was mostly within the range of 2–10 years, and in three studies, fathers of children aged 3–6 years were included [26, 30, 31]. Of the studies that specified the race or ethnicity of the participants, three comprised only Latinos or Mexican Americans [19, 27, 29], and one comprised only African Americans [32]; in the remaining studies, participants comprised different races and ethnicities. Most studies used thematic analysis for data analysis; however, three studies used the ground theory approach [27, 28, 32], and one used the content analysis method [30].

### Quality appraisal

Quality assessment scores were used to interpret the studies and synthesize their outcomes. Quality scores ranged from 7 to 8 (out of 10) (Table 1). As the quality of the study findings was consistently high across studies, we did not exclude any articles based on our quality assessment.

**Table 1:** Overall study description

Study (author, year, country)	Study purpose	Recruitment/inclusion criteria	Sample size	Interviewee	Age of children (range, mean)	Race/ ethnicity (%)	Data collection method	Analytic technique	Quality Appraisal
Harris, Jansen & Rossi, 2020 Australia [28]	To explore fathers' perceptions, beliefs, attitudes and lived experiences of mealtime interactions with children and other family members	Convenience sampling at a work site Fathers of children aged 0 –12 years	27	Fathers	$\le$ 12 years	Not specified	Focus group	Grounded theory	7
Khandpur, Charles, & Davison, 2016 USA [16]	To examine co-parenting dynamics in the context of food parenting in a diverse sample of fathers	Purposive sampling and snowball sampling from the community, existing database, social network Fathers of a child aged between 2–10 years	37	Fathers	2–10 years	White or black, non-Hispanic	semi-structured interviews	Thematic analysis	8
Khandpur, Charles, Blaine et al., 2016 USA [33]	To identify the specific food parenting practices utilized by fathers and describe how these practices varied by fathers’ education levels and their residential status	Purposive sampling and snowball sampling from the community, existing database, social network Fathers of a child aged between 2–10 years	40	Fathers	2–10 years	White & black	Semi-structured interviews	Thematic analysis	8
Lindsay et al. 2018 USA [19]	To examine Latino fathers’ beliefs, attitudes and practices related to their young children’s eating, physical activity, and sedentary behavior		28	Fathers	2 – 8 years	Latino	Focus group	Thematic analysis	7
Lowenstein et al 2013 USA [34]	To explore the cultural implications on Latino caregivers’ definition of obesity and feelings about the provider–parent interaction when dis cussing weight, diet and physical activity	Convenience sampling in the community Male parent/caregiver of a child who was 12 years or younger	24	Fathers	$\le$ 12 years	Black, White & Latino	Focus groups discussion; observation of verbal and nonverbal interactions	Thematic analysis	7
Neshteruk et al., 2020 [20]	To characterise fathers’ involvement in children’s physical activity as well as to identify the physical activity parenting practices commonly used by fathers	Convenience sampling in the community Fathers aged $\ge$ 18 years having a child aged between 3–11 years	24	Fathers	3 – 11 years	White, Black	Semi-structured interviews	Deductive thematic analysis	8
Salemonsen et al., 2022 [35]	to explore perceptions of contributing factors to childhood overweight and obesity among fathers of children with overweight or obesity and the fathers’ experiences of their parental role caring for a child with overweight or obesity	Purposive sampling in the community	8	Fathers	4–16 years	Not specified	Semi-structured interview	Thematic analysis	8
Sherman & Smith, 2019 USA [32]	To investigate African American (AA) fathers’ perceived role in promoting healthful decisions related to dietary patterns among their children	Purposive sampling with snowball sampling in the community AA fathers aged $\ge$ 18 years having a child aged between 6–18 years	20	Fathers	6 – 18 years	AA	Focus group	Grounded theory	8
Tan et al 2020 USA [26]	To understand how fathers and mothers jointly navigate child feeding and identify areas of child feeding where fathers and mothers agree and/or disagree	Convenience sampling in the community, clinics, child care centers etc married or cohabitating heterosexual couples, both have lived with the child for at least 1 years	30 couples = 60	Mothers, fathers	3– 5.99 years	White & black Non-Hispanic	Focus group	Thematic analysis	8
Turner et al 2014 USA [27]	To examine Mexican American and Mexican immigrant fathers’ and mothers’ approaches to making decisions about their family’s lifestyle behaviors, as well as current diet and physical activities	Convenience sampling in the Mexican community (local libraries and schools) Two parents Mexican–American or Mexican–immigrant families with school aged children	66	Mothers, fathers	1–18 years	Mexican- American & Mexican	Focus groups	Grounded theory	8
Vollmer 2018 USA [30]	To understand how fathers of preschool-age children define overweight and obesity in children and what leads a child to become overweight or obese, investigate how fathers control or attempt to control their child’s weight, and explore the father’s perceived role in making decisions regarding his child’s weight status	Online survey Fathers of $\ge$ one child aged 3–5 years reporting eating more than one meal/wk with the child, and being aged $\ge$ 18 years	117	Fathers	3–5 years	White, Non-Hispanic	Open-ended questions	Content analysis	7
Walsh et al. 2017 Australia [31]	To assess fathers' beliefs and perceived roles in the eating and physical activity behaviors of their young children, and examine fathers' views regarding the type of support they require to best promote healthy eating and physical activity behaviors in their young children	Purposive sampling and snowball sampling in the community Fathers of at least one child aged $\le$ 5 years	20	Fathers	Mean age = 3.6 years	Not specified	Semi-structured interviews	Thematic analysis	7
Zhang et al 2018 USA [29]	To explore Latino fathers’ perspectives and parenting practice experiences regarding early adolescents’ eating, physical activity, and screen-time behaviors	Convenience sampling at the Latino community centers and one charter school Fathers self-identified as Latino having a least one child aged 10–14 years	26	Fathers	10–14 years	Latino	Focus groups	Grounded theory	7

### Overarching themes

Three overarching themes were derived: (1) fathers’ parenting practices and role-modeling behaviors, (2) fathers’ roles in their relationships with other family members, and (3) fathers’ resource-seeking behaviors and contributions to their home food environment. These themes represent fathers’ perspectives on their influence on children’s health behaviors. Examples of quotes depicting each overarching theme and subtheme are presented in Table 2.

**Table 2:** Themes and example quote

Main theme	Sub-themes	Example quotes		
			Quotes	
father’s own parenting practices and role-modeling behaviors	fathers’ parenting practices	healthy	“Like now he wants something at night, we will let him have a bowl of cereal, but not a big bowl.... He is learning that he just can’t eat anything he wants, because he will get big.”	Turner 2014, p 566
			“I like to introduce new things to the kids once in a while and open up their minds to trying new stuff.”	Khandpur 2016, p 8
			"Yeah we always try to make sure we sit down together. I think that's pretty important. We'll all sit down and we'll make sure, yeah. There's no TV on, there's nothing like that, sort of just us."	Walsh 2017, p 6
			“We try and aim as a family, especially for meal time/dinner time to sit down as a family and share that as not only a time for eating but more so conversing and communicating with the family. So we see food as coupled with that as part of our rules, tradition that we like to sit down as a family.”	Walsh 2017, p 9
			“It [physical activity] is kind of my domain cause I feel like well I signed ‘em up.’”	Neshteruk et al., 2020, p 542
			“And it [physical activity]’s [boxing], like, an everyday routine, Monday through Friday. There’s sometimes he doesn’t like it; I know he doesn’t really, he gets tired, but I tell him it’s for your own good, you know. It’s for self-defense, healthy, it’s healthier, keep yourself busy. He lost a lot of weight, that’s what I like.”	Turner 2014, p 566
		unhealthy	“Now eat. I’ll sit at the table with him. You can’t leave this table until you eat.”	Khandpur 2016. P 9
			“If you don’t finish your dinner, you’re not gonna be able to have the dessert that I bought tonight.”	Khandpur 2016, p 9
			“Every morning before we put him on the bus, if he’s good, he gets a glazed donut.”	Khandpur 2016, p 9
			“if you eat this salad, I’ll give you a small piece of chocolate”	Zhang 2018, p 2074
			“I try to give him what he wants [ to eat]. He’s kind of spoiled”	Khandpur 2016, p 10
			“He’s looking at it[TV] the whole time. I feel like that keeps him focused.” -	Khandpur 2016, p 10
	fathers’ role-modeling behaviors	healthy	“I think it's extremely important. Your words, your actions, your behaviors have a great impact because I believe the home is the first school that they attend. So what they learn from us is eventually what their characters will be made up of.”	Walsh 2017, p 9
			"It's, you know, we're role models in all aspects of life and I think healthy eating habits and exercise and the whole package is such a critical part. I think there's a lot geared back to the parents taking responsibility for that because they're the ones setting the example of that sort of habits/culture. So yeah, no, it's a huge part of what we practice and preach."	Walsh 2017, p 6
			“I think it’s very important, you can tell your kids to eat vegetables but if you’re not eating them yourself it’s very hard. They think you’re not doing it so why should they. I think it’s very important.”	Walsh 2017, p 9
			"I mean I think kids learn by seeing and hearing. We try to model and it doesn't always work but we are conscious of leading by example for all the kids. (FA17). P6	Walsh 2017, p 6
			“I mean basic stuff, like we’d always encourage walking places that we can locally. I guess that also goes with your role modelling stuff and that we would always try and walk somewhere if we can, especially if it’s just a local activity”	Walsh 2017, p 10
			"I feel strongly that …I should lead by example. So whenever they're at soccer practice, I make sure that I'm there…running, exercising the entire time they are so they can see."	Neshteruk et al., 2020, p 542
		unhealthy	“Just recently I myself have worked to improve my health by not buying soda at the store. Falling back into bad habits [is a barrier]! Also, having an addiction to soda will be a barrier for me.”	Vollmer 2018, p 286
fathers’ roles in their relationships with their family members	Shared childcare and household responsibilities with spouses	healthy	“My wife and I split things pretty 50–50, right down the middle. She’s really in charge of Thursday, Friday and Saturdays, and I’m able to be there Monday, Tuesday, Wednesday. I do most of the cooking in the evenings when I’m at home. She takes the day, and I take the night.”	Khandpur 2016, p 458
			“Me and my wife, we try to split the responsibilities for cooking. As far as groceries go we both do it together. We don’t have a car. It makes it easier if we’re both together to help carry stuff in. I enjoy going shopping because I like to introduce new things to the kids once in a while and open up their minds a little bit to trying new stuff.”	Khandpur 2016, p 458
			“I’m definitely the primary parent with regards to grocery shopping. My wife is the primary parent on meal planning."	khandpur 2016, p 458
			“We do the same thing, we’ll do it at the same time. It’s not like she specifically does this and I specifically do that. Sometimes it’s a matter of who beats who to the kitchen. We’ll both cook for [our children] together”	khandpur 2016, p 458
		unhealthy	“My wife is the one that decides and she has everything prepared.”	Turner 2014, p 566
			“I leave it up to my wife. She is the one who is going to cook	Turner 2014, p 566
			‘She [mother] is most direct contact when she is buying the food and when she chooses what we eat	Lindsay et al., 2018, p 408
			“She’s the CEO (chief executive officer) of domestic duties right now, so she takes full ownership of that. Thank goodness.”	Tan 2020, p 4
	Similarity in the fathers’ and their spouses’ parenting practices	healthy	“Her mother’s on the same page with me when it comes to fast food, just really don’t do it much at all.”	Khandpur 2016, p 458
			“I think mealtime’s huge. Food draws us back together. It’s that centering point. We want that. My wife is good about that too and I just really try to support her in that.”	Khandpur 2016, p 458
			The most important thing is that parents cooperate and agree. It is also important to be flexible, be open about expectations and share the tasks. If we have agreed on a plan, it’s important that we stick to it	Salemonsen 2022, p 8
		unhealthy	“in my case my wife spoils them more,., I am a bit rougher, more imposing, and in the end, she is the one who has, let’s say control, over the child”	Zhang 2018, p 2074
			“I would take his game away and his mother would come and give it back to him"	Zhang 2018, p 2074
	Involvement of the children in activities	healthy	“I’ll say to my son, you’re gonna give me a hard time over vegetables today, so pick a vegetable you’ll eat today and we’ll go home and cook it.”	Khandpur 2016, p 7
			“… it's probably a bonding time actually for us like sitting down, eating, because it is one of the times that you are actually all together really … Yeah outside of your days off and stuff like that. Mealtimes would be the time that everybody is there.”	Harris 2020, p 3
			"As I think about my relationship with mys son, it's just good to spend time with your kids no matter what you're doing, but the physical activity thing adds another level of bonding.”	Neshteruk 2020, p 542
		unhealthy	"… Smartphones and the like have become a way of life for coming generations, with kids getting iPhones, iPods, iPads, and the like at a younger age with each generation. Kids no longer go outside and play or explore like they used to. They no longer get the activity that older generations used to get."	Vollmer 2018, p286
			“One day, I said to him, if you don't stop that iPad, I'm going to break that in front of you …. it's mainly communication, because we got told, you know, if you're around the table, you're supposed to face each other, talk to each other.”	Harris 2020, p 3
			“… like when the TV's on, he'll just be there, just blankly staring at it and he doesn't really interact. Once you turn the TV off, he'll have a little bit of a tantrum, but after that's past, he becomes really interactive and really funny …”	Harris 2020, p 4
			“they say no, we want food from outside”	Zhang 2018, p2074
fathers’ resource-seeking behaviors and contributions to their home food environment	seeking available resources	healthy	“I would consult the pediatrician and make sure they are eating a healthy, well-balanced diet to get their weight under control.”	Vollmer 2018, p286
		unhealthy	We live in a society where everything is on fast forward, where both parents are working full-time and are late home. This makes it easier to sometimes buy fastfood on your way home. We pay others to make our meals, no wonder we are gaining weight	Salemonsen 2022, p 6
			“I noticed eating healthy, it costs money, it’s kinda expensive, because you know, fresh, fresh, fresh, fresh, rather than frozen and pasteurized and so I think that maybe like with the kids, not say you know our kids, but kids in a community that don’t really have, their parents don’t have the funds, they get it where they buy the things that last longer and that’s not gonna be your fresh and healthy stuff.”	Sherman & Smith 2019, p 5
			"I guess people have less time so it's about food that's easy to prepare which is processed, which is typically like high in calories and low nutritional value."	Walsh 2017, p 6
	Contribution to their home food environment	healthy unhealthy	“We try to make a variety of food [available] as well.”	Harris 2020, p 4
			“Just recently I myself have worked to improve my health by not buying soda at the store. Falling back into bad habits [is a barrier]! Also, having an addiction to soda will be a barrier for me.”	Vollmer 2018, p286
			“Food companies know what it takes to make their food addictive and they’re incentivized to use this knowledge to sell as much as possible to everyone including families with kids. Parents are too tired, stressed, busy to fight it, and slowly they give in, their kids get addicted a vicious cycle ensues.”	Vollmer 2018, p286

### Fathers’ parenting practices and role-modeling behaviors

*Fathers’ parenting practices*, in this context, refer to behavioral strategies that guide their children’s health behaviors that relate to childhood obesity. The role-modeling behavior of the fathers involved exhibiting behavioral examples that influenced children through emulation. Fathers’ attitudes, beliefs, perceptions, and knowledge regarding diet and physical activity can shape their parenting practices and role-modeling behaviors that guide their children’s health behaviors.

### Fathers’ parenting practices

Fathers noted that their parenting practices may affect the healthy or unhealthy behaviors of children related to obesity in childhood. First, fathers reported certain parenting practices that could influence their children’s healthy behaviors. For example, fathers encouraged their children’s autonomy by letting them choose what they want to eat among various healthy options, respecting their children’s preferences, and negotiating with their children to guide them in choosing healthy options [27]. Fathers also introduced “new things to the kids” continuously, thus allowing their children to become familiar with diverse foods [18]. They also reported parenting practices with structural features, such as feeding on a schedule and setting rules regarding the eating environment (e.g., location of the meals, not watching television during meals, finishing a meal, types of food to be consumed, and the amount of food) [18]. Some fathers perceived mealtime as an important time for the family and considered food a means to bring the family together [31]. They also believed that mealtimes were a social event for helping children learn social skills [31]. Moreover, fathers indicated being in charge of their children’s physical activities; as one father stated, “It [physical activity] is my domain…” [20]. Fathers usually engage in setting a routine physical activity for their children [27].

Some fathers also exhibited certain parenting practices that could be considered unhealthy. For example, they pushed their children to eat more [18] and used food as a reward to entice their children to eat other foods or promote non-food-related behaviors, such as reducing television viewing or playtime [18, 29]. In such cases, fathers usually presented high-energy-dense sugary foods as a reward. Furthermore, some fathers recognized that their children were “spoiled”; for example, one father stated that he “tried to give him what he wants [to eat]” [18]. They also used media such as TV to distract their children while feeding them [18].

### Fathers’ role-modeling behaviors

Fathers were aware of their influence as role models. In addition to perceiving their critical roles in modeling good behaviors, they fathers recognized that they have a responsibility in shaping their children’s health behaviors [31]. Thus, fathers attempted to eat healthy foods, exercise more often, and avoided engaging in sedentary behaviors. A father stated, “[my] words, [my] actions, and [my] behaviors have a great impact” and “so, what they learn from us is eventually what will shape their character” [31]. A father stated, “They think you’re not doing it so why should they” [31]. Notably, the fathers indicated that they played a major role in influencing their children’s engagement in physical activities. Another father stated that he tried actively participating in physical activities because he thought his children would follow his behavior [31]. Another father attempted to accompany his children during their structured activities, “…running, exercising the entire time they are so they can see” [20].

However, fathers often failed to adhere to healthy behaviors because of their unhealthy dietary habits such as a high intake of soda, or because of their proclivity toward sedentary behavior [30]. Although these fathers were aware of their roles as models for their children, they had difficulty managing conflicts because of their preferences and behaviors.

### Fathers’ roles in their relationship with their family members

The fathers’ influence on children’s health behaviors was integrated with the nature of their relationships with their family members. The father’s role could be described based on whether they shared responsibilities in childcare and household work with their spouses rather than staying in their traditional role and whether their parenting practices were similar to those of their spouses. The fathers also identified encouraging children to be involved in various activities as a significant role.

### Shared childcare and household responsibilities with spouses

From the point of view of fathers, collaborative responsibility-sharing with their spouses emerged as a pivotal factor that influences their active involvement in parenting practices and, thereby, impacts their children’s health behaviors associated with obesity. Some fathers shared responsibilities with their spouses by taking charge of household work to promote their children’s healthy behaviors. One father stated that he and his wife split the work “right down the middle” [33]. The method of sharing responsibilities differed slightly across families. One father stated that he and his wife divided their household responsibilities, including cooking and childcare, based on the days of the week or hours of the day [33]. Another father reported that he oversaw specific tasks, such as grocery shopping, whereas she oversaw meal planning [33]. One father and his spouse worked together, doing the “same thing at the same time,” and indicated that they “both cook for [children] together” [33].

However, some fathers ascribed traditional gender roles to housework. They thought that cooking was their spouse’s responsibility; thus, they left it up to the spouse’s decision and practices [27]. One father indicated that household tasks were his spouse’s work by stating that “my wife is the one that decides and she has everything prepared” [36], and the menu is determined by what their spouse selects [19]. Another father reported that his “wife is the CEO of domestic duties” [26]. These fathers’ reluctance to participate in household tasks and childcare stems from their stereotypical perception of gender roles, which could impede their active engagement in parenting practices to promote children’s healthy behaviors.

### Similarity in the fathers’ and their spouses’ parenting practices

Fathers acknowledged that the congruence or discordance between their approaches and their spouses’ parenting practices significantly impacted their children's obesity-related behaviors. Some fathers adopted parenting practices similar to those of their spouses. A father reported that he and his spouse were “on the same page ‘about the diet option” [33]. One father stated that he supported his spouse’s thoughts and ideas regarding the importance of mealtime because they shared similar perspectives [33]. A father stated that two parents need to cooperate, share the tasks, and stick to the plan if both agree[35]. However, some fathers reported conflicts with their spouses regarding their parenting practices. For example, some fathers considered their spouses’ parenting practices to be unhealthy and thought that these practices influenced their children toward unhealthy behaviors [29]. One father reported that the children were “spoiled” because of how their mother fed them [29]. Regarding limiting screen time, a father stated that “I would take his game away, and his mother would come and give it back to him” [29].

### Involvement of the children in activities

Fathers interacted with their children and attempted to involve them in various activities, such as grocery shopping, eating together, and playing outside [28, 33]. Fathers considered these activities as “bonding time” with their children [27], noting that doing such activities with their children added “another level of bonding” [20] in their relationship. Fathers indicated that they played a significant role in their children’s physical activities; they were in charge of planning their children’s physical activities, and when they were active, they engaged in such activities with their children [20].

Fathers identified several challenges involving their children in their activities, including electronic media and children’s resistance. One father reported that “…smartphones and the like have become a way of life for the coming generations” [30]. Other media devices, such as televisions and iPads, have also been identified as challenges to fathers’ ability to encourage their children to be physically active [28]. The resistance of children to healthy options was another barrier identified. Fathers stated that it was a challenge impeding their children from choosing healthy options. One father reported that when he offered homemade food to his child, “they say no” and preferred to eat “food from outside” [29].

### Fathers’ resource-seeking behaviors and contributions toward their home food environment

Seeking the available resources and contributing to the physical home environment (i.e., the home food environment) were other important factors affecting fathers’ influence over their children’s health behaviors. Conversely, feeling frustrated owing to limited resources and challenging home food environments was identified as a challenge to improving their children’s health behaviors.

### Seeking available resources

Fathers sought available resources to support their children’s healthy behaviors but felt frustrated when they perceived a lack of sufficient resources. Some fathers reached out to their healthcare providers for valuable information and considered such information a resource to promote healthy behavior among children. One father stated, “I would consult the pediatrician and make sure they are eating a healthy, well-balanced diet to get their weight under control.” [30].

However, fathers perceived healthy food options as more expensive and were frustrated if they could not afford these options because of financial restrictions. One father reported, “I noticed eating healthy, it costs money, it’s kinda expensive” [32]. Furthermore, fathers highlighted time constraints when both parents work full time; this situation leads them to choose simple solutions, such as choosing fast foods for their children [35]. Fathers indicated that they did not have enough time to engage in healthy lifestyle choices by stating that “people have less time so it’s about food that’s easy to prepare, which is processed,” and they noticed that such options are “typically like high in calories and low nutritional value” [31].

### Contribution to their home food environment

Some fathers identified the home food environment as a facilitator of healthy food intake among their children. They purchased healthy foods to provide their children with healthy food choices. One father reported that “we try to make a variety of food [available]…” [28]. However, if the father preferred unhealthy foods, such as soda or high-calorie foods, they “fell into bad habits” and tended to have them in the house, potentially rendering such foods available to their children [30].

## Discussion

To date, there is a paucity of information on how to improve fathers’ role in addressing childhood obesity because fathers have been underrepresented in studies on childhood obesity. We contributed to meeting the knowledge and literature gap by synthesizing the findings of 13 qualitative studies to clarify paternal perspectives regarding their influence on children’s health behaviors. From the qualitative studies reviewed, we found evidence that fathers are aware of their influence on children’s health behaviors. Their parenting practices and role-modeling behaviors were among the most commonly addressed, consistent with known parental roles in children’s health behaviors [5]. Furthermore, fathers were cognizant of the importance of their relationships with other family members, which could be understood in terms of their role within the familial relationship. We suggest that these fathers’ perspectives be incorporated into observational studies to examine the relationship between fathers’ roles and children’s health behaviors to design further intervention programs to improve these behaviors.

We found that the analyses of fathers regarding the common parental role of influencing children’s eating habits and physical activities primarily focused on the related healthy or unhealthy parenting practices. Healthy parenting practices include respecting children’s autonomy in selecting healthy food options, introducing new foods to facilitate their familiarity with diverse foods, and setting structures around the mealtime routine. According to previous studies, both healthy eating behaviors and lower body mass index in children are associated with the frequency of family mealtimes [37, 38]. Our meta-analysis reveals that this relationship may be related to fathers’ healthy food-related parenting practices during mealtime. Reported unhealthy parenting practices included impelling children to eat more, using food as rewards to encourage certain behaviors, feeding children whatever they want, and using media as a tool to distract children while feeding. Such unhealthy parenting practices are consistent with those reported in earlier works on the factors influencing children’s unhealthy eating behaviors. According to a previous study, parenting practices, including parental pressure to eat, can disrupt the development of children’s self-regulation in response to hunger [39]. Additionally, children tend to be more responsive when parents use food as a reward [40].

Fathers also recognized that their behavior could serve as a model for their children. This is consistent with previous findings regarding the father as a role model for children’s behavior [41]. In the studies reviewed, physical activity was identified as the main behavior that fathers may model for their children. Some fathers recognized the benefits of physical activity and attempted to be more active in front of their children. This finding emphasizes fathers’ greater role in arranging physical activities than mothers [42]. Although fathers were aware of their role as models, some of them failed to adhere to healthy options because of their proclivity toward unhealthy behaviors. In a cross-sectional study of minority families, half of the participating parents exhibited unhealthy modeling behaviors, although the authors did not specify which parents’ role-modeling behaviors were associated with children’s health behaviors [43]. This finding indicates that adults may need to improve their health behaviors to improve their health as well as encourage their children’s healthy behaviors.

A notable perspective identified in the present meta-synthesis was fathers’ perception of their role in their relationships with family members. Their role was described in terms of their participation in childcare and household work, specifically, whether they worked with their spouses and children. Some fathers were more likely to share childcare and household responsibilities with their spouses than others were. Traditionally, fathers are viewed as additional caregivers or supporters of the primary caregiver; in the studies reviewed, fathers’ perceptions varied from active engagement in childcare by interacting with their spouses to maintenance of the traditional gender role in the family.

Shared parenting responsibilities and similarities in healthy parenting practices lead to positive cognitive [44] and behavioral [45] outcomes among children. Consistent with these findings, taking responsibility for childcare and performing parenting practices similar to those of spouses could be important factors affecting children’s health behaviors. However, according to one previous study, fathers and mothers could have different parenting practices with different influences on their children’s health behaviors [7]. For example, the researchers found that, compared to fathers, mothers were more likely to use limit setting (e.g., limiting weekly television and videogame time) and monitoring (e.g., keeping track of the amount of exercise the child received) and less likely to use control (e.g., ensuring that children always finished the food on their plate) [7]. The differences between the parenting practices of fathers and mothers may be understood in the context of fathers’ perceptions of their role in their spousal relationship, as observed in the studies reviewed in this meta-synthesis.

Fathers also indicated the importance of interacting with their children. In the reviewed studies, some fathers perceived engaging in activities with their children as an opportunity to build relationships with them, and the degree of closeness between fathers and their children is positively associated with children’s body mass index [46]. Thus, interactions between fathers and children during certain activities, including eating and physical activities, may be an important factor for improving children’s health behaviors [47]. In our meta-synthesis, some fathers reported that they attempted to involve their children in grocery shopping and physical activities and felt connected with their children during this time. Consistent with this finding, fathers’ perspectives on the extent to which they participate in childcare are related to their positive influence on children’s health behaviors [48].

Seeking available resources and contributing to the physical home environment were identified as factors influencing children’s health behaviors. The availability of the right resources is known to influence children’s health behaviors and childhood obesity [36, 49]. Fathers’ active resource-seeking behaviors could be a potential factor in improving children’s health behaviors. Prior research has also found that the physical home food environment (i.e., availability of homemade food) is a significant factor affecting children’s dietary patterns [50, 51]. Children develop their dietary patterns and food preferences based on what is available in their home food environment as they observe their parents’ preparation and selection of foods [52]. In studies reviewed, many fathers contributed to establishing the home food environment by purchasing healthy foods to provide healthy food choices to their children. However, fathers who preferred to consume soda or high-calorie foods tended to stock them at home, which also made these unhealthy options available to their children.

### Limitations

There are some limitations in interpreting the outcomes of the present study. In four of the 13 studies, fathers from Hispanic or African American families were recruited; thus, the role of family members should be understood from their cultural perspective. Furthermore, given the heterogeneity of the samples (e.g., child age range and racial or ethnic composition), different study methodologies (e.g., data collection approach and analysis methods), and the overall diversity of the included primary studies, it was challenging to synthesize the study findings. Finally, our process of retrieving the relevant literature may not have been comprehensive because we included only peer-reviewed journals.

### Implications

Despite the limitations of this study, our qualitative meta-synthesis provides potential recommendations for nursing practice, research, and policy. In nursing education, nurses may need to emphasize the role of fathers in improving children’s health behaviors. Thus, fathers would be aware of their significant influence on children’s health behaviors and actively participate in childcare by working with their spouses. Although several fathers recognized their influence on children’s health behaviors, mothers and fathers may share different perspectives on their respective parenting roles.

In future nursing research, researchers may consider comparing the perceptions of fathers’ and mothers’ influences on their children’s health behaviors using diverse approaches, such as objective measures of behaviors. Furthermore, more research is needed to understand how fathers can actively engage in childcare and define how parents can work together to encourage fathers’ active participation in childcare and household work. Moreover, few studies have targeted fathers of adolescents, and more research is needed to explore the influence of fathers of adolescents on children’s health behaviors. The fathers’ perspectives on their influence on children’s health behaviors identified in this study could be used to develop interventions to improve fathers’ parenting practices, role-modeling behaviors, and relationships with their spouses and children. Therefore, fathers could be included in family-based interventions to reduce childhood obesity. Intervention research focusing on fathers’ role in establishing children’s healthy behaviors could be conducted in a way that encourages joint activities among family members, such as physical or fun cooking activities [53, 54]. Furthermore, increasing the number of paternity leaves could be a policy measure to provide fathers with the opportunity to actively participate in parenting practices and spend more time with their families.

## Conclusion

We synthesized 13 qualitative studies to understand fathers’ perspectives regarding their influence on children’s health behaviors related to childhood obesity. Through a qualitative meta-synthesis, we found that fathers were aware of their parenting practice role-modeling behaviors that could influence their children’s health behaviors. In addition, fathers' perspectives on their influence over their children’s health behaviors could be understood in terms of their relationships with their spouses and other children; that is, whether they shared household and childcare responsibilities with their spouses and involved their children in various activities. Based on the findings of this review, nursing researchers would further investigate possible approaches for improving fathers’ roles in establishing children’s healthy behaviors—particularly the behaviors related to obesity—with an understanding of the family dynamics in a cultural context.

### Supplementary Information

**Additional file 1.**

**Additional file 2.**

## Data Availability

The datasets used and/or analyzed during the current study are available from the corresponding author upon reasonable request.

## Abbreviations

ENTREQ: Enhancing transparency in reporting the synthesis of qualitative research
PRISMA: Preferred Reporting Items for Systematic Reviews and Meta-Analyses
